# The William F. Jenks Memorial Prize

**Published:** 1895-09

**Authors:** 


### The William F. Jenks Memorial Prize.

This Prize, the competition for which we announced in June, 1894, has been awarded to A. Brothers, M.D., 162 Madison Street, New York, for the best essay on "Infant Mortality During Labour, and its Prevention."

